# Does cardiorespiratory fitness moderate the relationship between overweight, cardiovascular risk markers and mental health among forcibly displaced individuals living in a Greek refugee camp?

**DOI:** 10.3389/fspor.2024.1334230

**Published:** 2024-10-21

**Authors:** Markus Gerber, Konstantinia Filippou, Florian Knappe, Ioannis D. Morres, Emmanouil Tzormpatzakis, Elsa Havas, Harald Seelig, Flora Colledge, Sebastian Ludyga, Marianne Meier, Yannis Theodorakis, Roland von Känel, Uwe Pühse, Antonis Hatzigeorgiadis

**Affiliations:** ^1^ Department of Sport, Exercise and Health, University of Basel, Basel, Switzerland; ^2^ Department of Physical Education and Sport Sciences, University of Thessaly, Trikala, Greece; ^3^ Department of Nutrition and Dietetics, University of Thessaly, Trikala, Greece; ^4^ Department of Health Sciences and Medicine, University of Lucerne, Lucerne, Switzerland; ^5^ Interdisciplinary Center for Gender Studies, University of Bern, Bern, Switzerland; ^6^ Department of Consultation-Liaison Psychiatry and Psychosomatic Medicine, University Hospital Zurich, University of Zurich, Zurich, Switzerland

**Keywords:** cardiovascular risk factors, fitness, mental health, moderation, refugees

## Abstract

**Objectives:**

Refugees may have an increased risk of developing overweight/obesity as they often experience a nutritional transition. Because maintaining good cardiorespiratory fitness can help reduce the negative impact of excess weight on overall health, the objective of this study was to examine whether fitness moderates the relationship between weight status and cardiovascular and mental health outcomes in forcibly displaced individuals living in a Greek refugee camp.

**Methods:**

A sample of 142 forcibly displaced individuals were recruited. Cardiorespiratory fitness was assessed with the submaximal Åstrand-Rhyming bicycle ergometer test. Blood pressure, blood lipids, blood glucose, and hs-CRP were assessed as physical health outcomes, whereas post-traumatic stress disorder, depression and anxiety symptoms, pain, and quality of life were assessed as mental health outcomes. Main and interaction effects were tested via analyses of covariance (ANCOVAs).

**Results:**

Almost 50% of the participants were overweight/obese, more than 60% presented with very poor fitness levels, and the percentage of participants with very poor fitness levels was particularly high among overweight/obese participants. Whereas overweight/obesity was associated with a less favorable body composition and cardiovascular risk profile, poor fitness was associated with a higher percentage of body fat and a lower percentage of muscle mass. Cardiorespiratory fitness did not moderate the relationship between overweight/obesity and most of the assessed health outcomes.

**Conclusions:**

Only limited support was found for the applicability of the fit-but-fat concept to our population of forcibly displaced individuals. Public health services should prioritize measures to prevent overweight/obesity and associated diseases in refugee camps. Moreover, efforts are needed to improve the fitness of camp residents via exercise/sport interventions.

## 1 Introduction

Refugees often face numerous challenges related to healthy nutrition (1), limited access to healthcare (2), and disrupted living conditions (3), which can contribute to the development of overweight and obesity (4). As highlighted by the United Nations Organization (5), safeguarding the health of refugees and migrants is a vital part of achieving sustainable development goal (SDG) 3 to ensure healthy lives and promote well-being for all at all ages. In a recent meta-analysis, the global prevalence rates of overweight and obesity in refugees were estimated to be 29% and 23%, respectively (6). However, the prevalence of overweight and obesity among refugees can vary depending on various factors such as their age, sex, region of origin, living conditions, and length of time spent in displacement (7–9).

Refugees may have an increased risk of developing overweight and obesity as they often experience a nutritional transition when they move from their home countries to a host country (10). Refugees often come from regions with different food cultures and availability (11). When they arrive in a new country, they may find a different range of foods, including those that were not commonly consumed in their home countries. This can lead to changes in their dietary habits as they adapt to the available food options (12). Refugees may also face challenges in adapting to the local food culture in their host countries. While some may try to maintain their traditional dietary practices, others may find it necessary to modify their diets due to the lack of familiar ingredients or cooking facilities (13, 14). In addition, refugees often face economic challenges upon arrival in a new country (15). Limited financial resources may restrict their access to a varied and balanced diet (11). In some cases, refugees may rely heavily on low-cost and processed foods, which can be high in calories, but lacking in essential nutrients (16). In this regard, studies have highlighted that overweight and obese individuals can still suffer from nutritional deficiencies if they consume energy-dense, nutrient-poor foods (17, 18). This can lead to an increased risk of malnutrition and related health problems (19).

As in any population (20), studies in different refugee and migrant populations have shown that overweight and obesity can have detrimental health implications (21, 22). Previous research with refugees revealed that overweight and obesity were associated with a higher risk of developing chronic diseases, including cardiovascular diseases (23), type 2 diabetes (24), and hypertension (25). Prior research has also shown that overweight and obesity can contribute to poor self-esteem (26), body image issues (27), and increased risk of mental health conditions such as depression and anxiety (28, 29). Refugees may already face high levels of psychological stress (30), and weight-related concerns can exacerbate their mental health difficulties (31). Conversely, mental distress can lead to increased food intake and contribute to weight gain (32). In addition, excess weight can strain joints, leading to musculoskeletal problems (33), including pain, arthritis, and mobility issues. Overweight and obese individuals may also encounter reduced physical mobility (34), resulting in a decreased capacity to engage in daily activities and may potentially limit their participation in social and community life within a refugee camp (including participation in physical activity, exercise, and sport) (35, 36). Finally, overweight and obese refugees may face challenges in accessing appropriate healthcare, as the healthcare systems in refugee camps may be overwhelmed, and thus lack resources to effectively address weight-related issues (37–39). It has therefore been concluded that addressing overweight and obesity should be *a priori*ty in refugees, with a focus on nutrition education, access to healthcare services, and physical activity programs (40, 41).

One advantage of promoting regular physical activity might be that it increases cardiorespiratory fitness (42, 43), which has been shown to mitigate the effects of overweight on health (44). While being overweight or obese is generally associated with an increased risk of various health conditions (as highlighted above), maintaining good cardiorespiratory fitness can help reduce the negative impact of excess weight on overall health (45). Cardiorespiratory fitness refers to the ability of the cardiovascular and respiratory systems to supply oxygen to the muscles during physical activity (46). Regular physical activity and aerobic exercise, such as brisk walking, jogging, or cycling, improve cardiorespiratory fitness (47, 48). Participation in aerobic exercise can moderate the effects of overweight on health by (a) lowering blood pressure, reducing the risk of cardiovascular disease, and improving overall cardiovascular health (49); (b) enhancing insulin sensitivity and glucose metabolism, which can help individuals with excess weight or obesity manage their blood sugar levels more effectively and reduce the risk of developing type 2 diabetes (50); (c) contributing to weight loss or weight maintenance, by increasing metabolism and promoting the preservation of lean muscle mass, which is beneficial for overweight individuals (51); (d) strengthening the respiratory muscles and enhancing lung capacity (52); and (e) reducing stress and improving mental health (53, 54). In the scientific literature, this positive effect of aerobic exercise in the overweight and obese is also known as the “fitness-fatness paradox” (55). It refers to the observation that individuals who are overweight or obese may nevertheless exhibit signs of good cardiorespiratory fitness. Traditionally, body mass index (BMI) has been widely used as a measure of body fatness and health (56). BMI is calculated by dividing a person's weight (in kilograms) by the square of their height (in meters). However, BMI does not differentiate between fat and lean mass (muscle, bone, and organs), nor does it account for differences in body composition (57). The fitness-fatness paradox also suggests that good cardiorespiratory fitness can be achieved independent of the degree of overweight and obesity (58). Nevertheless, it is important to recognize that the fitness-fatness paradox does not imply that excess body weight is harmless. However, the paradox emphasizes the importance of not relying solely on weight or BMI as predictors of overall health, but adopting a more comprehensive and nuanced approach by also looking at participants’ cardiorespiratory fitness (59).

Previous studies have shown that forcibly displaced individuals living in refugee camps often have lower fitness levels than the general population (60). During their flight or daily life in a refugee camp, refugees often face a range of challenges that can affect their cardiorespiratory fitness levels (61, 62). Such challenges include a disruption of physical activity routines (e.g., lack access to safe and appropriate spaces for physical activity, legal barriers to work), restricted access to healthcare (e.g., preventive care and treatment for chronic conditions), inadequate nutrition, mental health issues (with an indirect impact on cardiorespiratory fitness by affecting motivation, or energy levels), as well as environmental factors (e.g., exposure to poor air quality, extreme temperatures) (63, 64).

Given this background, the aims of our study were as follows: First, to assess the prevalence of overweight and obesity in a sample of forcibly displaced individuals living in a Greek refugee camp. Second, to assess participants cardiorespiratory fitness levels. Third, to examine whether and to what degree (a) normal weight and overweight/obese participants and (b) participants with low vs. higher fitness levels differ in body composition, cardiovascular risk markers, and mental health outcomes. Fourth, to examine whether participants’ fitness levels moderate the relationship between their weight status and cardiovascular and mental health outcomes. The present study will make an important contribution to the existing body of research since there is limited knowledge about the cardiorespiratory fitness level of residents in refugee camps. Moreover, it is unclear so far whether the “fit but fat” concept can also be observed in forcibly displaced individuals.

## 2 Methods

### 2.1 Participants and procedures

The data come from a larger pragmatic randomized controlled trial (RCT) (blinded). In this paper, cross-sectional baseline data are presented. Ethical approval was obtained from the local ethical review boards (Greece: IEC-DPESS 1701 [Internal ethics committee of the Department of Physical Education and Sport Science, University of xxx (blinded)]; Switzerland: EKNZ AO_2020–00036 [Ethic commission of Northwestern and Central Switzerland]). The study took place in a remote area in Central Greece in a refugee camp, which is under the governance of the Ministry of Migration and Asylum. In the camp, residents live in containers (equipped with a bathroom and kitchen), either together with family members or with a maximum of four individuals of the same sex and origin. The residents spend most of their time in the camp. Due to legal barriers, they are not allowed to work. Leisure activities are scarce, and the remote location makes it difficult to escape the camp's daily routine.

Participants were eligible if they met the following inclusion criteria: (a) living in the selected refugee camp, (b) 16–59 years old, (c) able to read in English, Arabic, Farsi, or French, and (d) provided written informed consent. We decided not to include children and younger adolescents to ensure that participants have the cognitive capacity to understand the questions included in the questionnaires. For pragmatic reasons, we also decided not to include older adults (≥60 years) to reduce the heterogeneity in the sample. Including children/younger adolescents and older adults would have required us to offer more exercise and sport activities that meet the specific preferences/needs of these target populations. Parental/guardian informed consent for minors (16–18 years) was not needed in the present project, because according to Swiss laws (Federal Act on Research involving Human Beings, HRA, Art. 23, 1a), legal representatives only need to provide written informed consent for their adolescent child (aged 14–17 years) if a project entails more than minimal risks and burdens (which was not the case for the present study). A screening was performed with potentially eligible households to draw a random sample stratified by sex. For the RCT, the minimal estimated sample size to demonstrate an intervention effect on post-traumatic stress disorder (PTSD) symptoms (the primary outcome of this RCT) was 136 participants (65).

The screening, recruitment, and data assessment took place in May 2021 by the research team together with 10 trained research assistants who were familiar with the residents’ cultural background and the camp's contextual setting. Both written and verbal information were given to the participants in their native languages, and participants provided written informed consent before the first data assessment. All participants were assured that participation is voluntary, and that withdrawal is possible at any time without any disadvantages, particularly with regard to the asylum process. Data assessment was carried out at the nearby Department of Physical Education and Sport Science of the University of Thessaly (65). Participants received information about their results after completion of the assessment and were referred to a specialist in case of a potential health risk. As further incentives, participants received a meal and some sport equipment.

### 2.2 Measures

Due to space constraints, only a concise description of the applied instruments is given here. More detailed information can be found in the published study protocol (65).

Body weight and body composition were assessed with a digital weighing scale (BC-545, Tanita, USA) that also allowed bioelectrical impedance analysis to measure the percentage of body fat and muscle mass. Body height was assessed with a stadiometer. Blood pressure was assessed three times (with a 2-min interval) with an Omron® digital blood pressure monitor after participants had rested for 5 min in a seated position (66). Finger prick methodology was applied to obtain capillary blood samples. Total cholesterol, low- (LDL) and high-density (HDL) lipoprotein cholesterol, triglycerides, and blood glucose levels (HbA1c) were analysed via Afinion 2 analysers (Abbott, Wädenswil, Switzerland). Afinion 2 point-of-care (PAC) analyser results correspond well with laboratory tests for both lipid levels and HbA1c (67, 68). In addition, 20 μm blood were collected with a Minivette® POCT EDTA (Sarstedt AG, Nümbrecht, Germany) to assess high-sensitivity C-reactive protein (hs-CRP). Analyses were done in a Cube-S Eurolyser device (Eurolyser Diagnostica GmbH, Salzburg, Austria) (69).

We applied the Åstrand-Rhyming Indirect Test of Maximal Oxygen Uptake (70), a submaximal bicycle ergometer test, to measure participants’ cardiorespiratory fitness. Based on sex, a correction factor for age, body weight, mean steady state, and power output, oxygen uptake as peak VO_2_max (ml/kg/min) was calculated (71). This test has been used previously in studies on refugees (62).

All psychological measures were assessed with instruments that have been previously employed in studies involving forcibly displaced adults (62, 72–74). Given that many participants had limited English skills, the questionnaires were available in English, Arabic, Farsi, and French language, and translators supported the data assessment process. All instruments have been previously validated in English, Arabic, Farsi, and French (65), and the scales had acceptable or good internal consistency (Cronbach's alpha >0.7) in our pilot study (61). PTSD symptoms were measured with the 22-item Impact of Event Scale-Revised (IES-R) (75), which refers to DSM-5 (76) and ICD-10 (77) criteria of PTSD. Answers were given on a five-point Likert scale from 0 (not at all) to 4 (extremely), resulting in an overall score between 0 and 88 points. Depressive symptoms were measured with the 9-item Patient Health Questionnaire (PHQ-9) (78), which refers to DSM-5 criteria for major depression (76). Answers were given on a four-point Likert scale from 0 (not at all) to 3 (nearly every day), with overall scores varying between 0 and 27. Anxiety symptoms were measured with the 7-item General Anxiety Disorder scale (GAD-7) (79), which refers to DSM-5 criteria for generalized anxiety disorder (76). Answers were given on a four-point Likert scale from 0 (not at all) to 3 (nearly every day), with overall scores ranging between 0 and 21. Pain was measured with the 5-item Visual Analogue Scale for Pain (VAS) (80), which asked for pain over the last week in the head, back, chest, stomach, and extremities (arms, legs, hands, feet). Item scores ranged from 0 (no pain) to 100 (pain as bad as it could be) and the average was calculated, to build a total index ranging from 0 to 100. Quality of life was measured with the 5-item World Health Organization (WHO) Index (81). Answers were given on a Likert scale ranging from 0 (at no time) to 5 (all the time). The individual item scores were summed, yielding an overall score between 0 and 25 (82).

### 2.3 Statistical analyses

Descriptive statistics (*n*, %, *M*, *SD*, *Min*, *Max*, *Skew*, *Kurt*) are presented to describe the characteristics of the sample and distribution of the study variables. Before carrying out the main analyses, data were screened for univariate outliers (>3.5 *SD* above the mean). Univariate outliers were excluded from all further analyses. If substantial deviations from normality (defined as *Skew* >2 and/or *Kurt* >7) (83) were observed after exclusion of univariate outliers, these variables were log-transformed before carrying out any further analyses. For mental health outcomes, the internal consistency was tested via Cronbach's alpha, which was expected to be ≥.70. Chi^2^-tests (*χ*^2^) and one-way analyses of covariance (ANCOVAs) were used to test differences between participants classified as normal weight and overweight/obese (due to the limited sample size, overweight and obese participants were combined in one group). Participants were classified as overweight if their BMI was ≥25 kg/m^2^ and as obese if their BMI was ≥30 BMI kg/m^2^. One-way ANCOVAs were applied to test differences between participants with different fitness levels. Classification of fitness levels are based on standards of the American College of Sports Medicine, adjusted for participants’ age and sex (84). Finally, a series of two-way ANCOVAs was used to test the interaction (moderator effect) between weight and fitness status. In these analyses, weight status and cardiorespiratory fitness were used as fixed factors (including an interaction term). Age, sex, education, time away from home country and time living in the refugee camp were considered as potential confounders. All analyses were carried out with SPSS version 28 for Mac (IBM Corporation, Armonk, USA), and the level of significance was set at *p* < 0.05 across all analyses. Effect sizes were interpreted as follows: small: *η*^2^ ≥ .01, medium: *η*^2^ ≥ .06, and large: *η*^2^ ≥ .138 (85).

## 3 Results

### 3.1 Sample characteristics

In total, 150 participants (76 men, 74 women) presented with valid BMI values. Of these participants, 56.7% (*n* = 85) completed the questionnaire in Farsi, 22.7% (*n* = 34) in Arabic, 14.7% (*n* = 22) in French, and 6.0% (*n* = 9) in English. The majority of the participants came from Afghanistan (*n* = 73), Somalia (*n* = 22), Congo (*n* = 18), or Syria (*n* = 12). Other reported home countries were Iran (*n* = 8), Iraq (*n* = 3), Sierra Leone (*n* = 2), Turkey (*n* = 2), Cameroon (*n* = 1), Guinea (*n* = 1), and Pakistan (*n* = 1) (7 participants did not answer this question). Concerning educational background, 37 participants reported not having any formal education, 51 completed primary school, 34 high school, and 20 completed higher education (university) (8 participants did not answer this question). Given the low number of underweight participants, we decided to exclude these participants from all further comparisons, resulting in a final sample of *n* = 144 participants (73 men, 71 women).

### 3.2 Descriptive results

With regard to weight status, 4.0% were classified as underweight (*n* = 6; 3 women, 3 men), 48.0% were normal weight (*n* = 72, 45 men, 27 women), 29.3% were overweight (*n* = 44, 22 men, 22 women), and 18.7% were obese (*n* = 28, 6 men, 22 women). With regard to cardiorespiratory fitness (estimated VO_2_max), 62.1% (*n* = 72) of the participants presented with very low fitness levels (1–19 percentile), 15.5% (*n* = 18) with poor levels (20–39 percentile), 9.5% (*n* = 11) with fair levels (40–59 percentile), 5.2% (*n* = 6) with good levels (60–79 percentiles), 4.3% (*n* = 5) with excellent levels (80–94 percentiles), and 3.4% (*n* = 4) with superior levels (≥95 percentile). Since more than six out of ten participants were classified in the lowest fitness category, in all subsequent analyses, we compared this group to their counterparts with higher fitness levels.

Means, standard deviations, minimal and maximal scores, skewness and kurtosis are reported in Table 1 for all study variables. For HbA1c and hsCRP, 3 and 7 cases were identified as univariate outliers, respectively. Because HbA1c and hsCRP still showed major deviations from normality after exclusion of outliers, these variables were log-transformed. As shown in Table 1, internal consistency (Cronbach's alpha) was satisfactory for all mental health outcomes.

**Table 1:** Descriptive statistics.

	*N* ^a^	*M*	*SD*	*Min*	*Max*	* α^b^*	*Skew*	*Kurt*
Sociodemographic background								
Age (years)	138	29.35	9.29	16	58	—	0.96	0.64
Months away from home country	125	33.72	35.28	2	300	—	4.79	29.72
Months in Koutsochero refugee camp	133	14.59	10.23	0	48	—	0.71	−0.13
Fitness								
Estimated VO_2_max (ml/kg/min)	116	32.08	68.87	9.05	68.87	—	0.35	0.25
Body composition								
Height (m)	144	1.64	0.10	1.41	1.93	—	0.21	−0.26
Weight (kg)	144	70.20	14.49	40.0	117.8	—	0.52	0.40
Waist (cm)	144	88.14	14.74	48	131	—	−0.16	0.03
Body mass index (kg/m^2^)	144	26.10	5.02	18.71	42.44	—	0.99	0.84
Fat mass (%)	143	29.10	11.07	9.30	56.60	—	0.33	−0.83
Muscle mass (%)	137	66.98	10.61	41.23	86.12	—	−0.27	−0.87
Cardiovascular risk markers								
Systolic blood pressure (mm HG)	143	120.43	12.97	89	156	—	0.33	0.06
Diastolic blood pressure (mm HG)	143	81.82	8.50	59.67	107.67	—	0.43	0.30
Total cholesterol (mmol/L)	129	4.11	0.92	1.92	6.61	—	0.39	−0.06
LDL cholesterol (mmol/L)	128	2.21	0.73	0.53	4.29	—	0.29	−0.28
HDL cholesterol (mmol/L)	129	1.18	0.31	0.51	1.95	—	0.42	−0.25
Triglycerides (mmol/L)	129	1.66	0.82	0.56	5.18	—	1.24	2.12
HbA1c (%)	135	5.41	0.38	4.70	7.40	—	2.53	10.47
hsCRP (mg/L)	129	1.71	1.58	0.60	9.17	—	2.20	10.47
Mental health								
PTSD symptoms (IES-R)	142	35.11	22.20	0	79	.94	0.09	−1.07
Depressive symptoms (PHQ-9)	143	10.67	7.27	0	26	.86	0.07	-1.08
Anxiety symptoms (GAD-7)	134	9.24	6.51	0	21	.90	0.18	-1.15
Pain (VAS)	132	26.47	21.73	0	95	.77	0.86	0.29
Quality of life (WHO-5)	142	13.73	7.11	0	25	.88	−1.00	−1.03

VO_2_max, maximal oxygen uptake; LDL, low density lipoprotein; HDL, high density lipoprotein; HbA1c, glycated hemoglobin A1c; hsCRP, high-sensitivity C-reactive protein; IES-R, 22-item impact of Event Scale—revised; PHQ-9, 9-item depression scale of the patient health questionnaire; GAD-7, 7-item general anxiety disorder scale; VAS, 5-item visual analogue scale; WHO-5, 5-item quality of life index of the World Health Organization.

^a^ Variations in *N* due to different number of missings for varying outcomes.

^b^ Cronbach's alpha.

### 3.3 Association of weight status and cardiorespiratory fitness with covariates

Compared to men (38.4%, *n* = 28), women were more likely to be overweight/obese (62.0%, *n* = 44), *χ*^2^(1,144) = 8.03, *p* < 0.01. No significant association was found for education (*p* > 0.05). Overweight/obese participants were older (*M* = 32.77 ± 9.75 years) than their normal weight counterparts (*M* = 25.93 ± 7.42 years), *F*(1,137) = 21.51, *p* < 0.001, *η*^2^ = 0.137, but did not differ in time away from their home country or time living in the Koutsochero refugee camp (*p* > 0.05).

Compared to men (53.1%, *n* = 34), women (73.1%, *n* = 38) were overrepresented among participants with very low fitness levels, *χ*^2^(1,116) = 4.85, *p* < 0.05. By contrast, no significant associations were found between cardiorespiratory fitness and education, age, months away from home country, and months living in the Koutsochero refugee camp (all *p* > 0.05).

### 3.4 Differences in health outcomes based on participants’ weight status

Table 2 shows that after controlling for age and sex, normal weight and overweight/obese participants differed in several of the assessed variables. While differences were observed in cardiorespiratory fitness, body composition, and most of the cardiovascular risk markers, no differences were found for any of the mental health outcomes. More specifically, overweight/obese participants had lower estimated VO_2_max (small effect), higher fat mass (large effect), lower muscle mass (large effect), higher systolic and diastolic blood pressure (small effect), higher total cholesterol (medium effect), higher LDL cholesterol (medium effect), higher HbA1c (small effect) and higher hsCRP (small effect). For anxiety and pain, initially significant group differences between normal weight and overweight/obese participants disappeared after controlling for covariates.

**Table 2:** One-way analyses of covariance (controlled for age and sex) with weight status as fixed factor, and cardiorespiratory fitness, body composition, cardiovascular risk markers, and mental health as outcome variables.

	Normal weight (*n* = 72)		Overweight/Obese (*n* = 72)			
	*M*	*SD*	*M*	*SD*	*F*	*η* ^2^
Cardiorespiratory fitness						
Estimated VO_2_max (ml/kg/min)	35.89	10.68	17.71	9.91	4.75*	.041
Body composition						
Fat mass (%)	22.52	8.09	35.94	9.93	82.97***	.384
Muscle mass (%)	73.40	8.01	60.84	9.47	77.94***	.380
Cardiovascular risk markers						
Systolic blood pressure (mm HG)	118.98	13.06	121.52	13.18	4.48*	.033
Diastolic blood pressure (mm HG)	79.72	7.64	83.38	8.81	6.20*	.045
Total cholesterol (mmol/L)	3.81	0.84	4.37	0.89	9.02***	.070
LDL cholesterol (mmol/L)	2.02	0.59	2.38	0.77	8.30**	.066
HDL cholesterol (mmol/L)	1.21	0.30	1.17	0.34	2.30	.019
Triglycerides (mmol/L)	1.46	0.74	1.82	0.82	2.96	.024
HbA1c (%)	5.29	0.22	5.52	0.47	6.08*	.046
hsCRP (mg/L)	1.36	1.55	2.07	1.60	5.22*	.042
Mental health						
PTSD symptoms (IES-R)	33.60	21.31	37.02	22.67	0.18	.001
Depressive symptoms (PHQ-9)	9.81	7.00	11.61	7.51	0.03	.000
Anxiety symptoms (GAD-7)	8.22	6.04	10.54	6.85	0.01	.000
Pain (VAS)	22.69	21.17	31.36	21.08	0.23	.002
Quality of life (WHO-5)	13.82	7.13	13.58	7.13	0.34	.003

VO_2_max, maximal oxygen uptake; LDL, low density lipoprotein; HDL, high density lipoprotein; HbA1c, glycated hemoglobin A1c; hsCRP, high-sensitivity C-reactive protein. IES-R, 22-item impact of event scale—revised; PHQ-9, 9-item depression scale of the patient health questionnaire; GAD-7, 7-item general anxiety disorder scale; VAS, 5-item visual analogue scale; WHO-5, 5-item quality of life index of the World Health Organization.

* *p* < 0.05.

** *p* < 0.01.

*** *p* < 0.001.

### 3.5 Differences in health outcomes based on participants’ fitness levels

As mentioned above, 62.1% (*n* = 72) of the participants presented with very low fitness levels (1–19 percentile). When these participants were compared to their fitter counterparts, differences were found in body composition and hsCRP (Table 3). More specifically, participants with very poor fitness levels had higher BMI (medium effect), higher fat mass (large effect), lower muscle mass (large effect), and higher hsCRP (medium effect). No significant differences were found in the remaining cardiovascular parameters and in any mental health outcomes.

**Table 3:** One-way analyses of variance (controlled for age and sex) with fitness status as fixed factor, and fitness, body composition, cardiovascular risk markers, and mental health as outcome variables.

	Very poor fitness (*n* = 72)		Higher fitness (*n* = 44)			
	*M*	*SD*	*M*	*SD*	*F*	η^2^
Cardiorespiratory fitness						
Estimated VO_2_max (ml/kg/min)	25.63	7.22	42.64	7.62	155.77***	.582
Body composition						
Body mass index (kg/m^2^)	26.79	5.07	23.46	3.09	8.87**	.073
Fat mass (%)	31.34	10.89	22.14	9.00	17.99***	.138
Muscle mass (%)	64.88	10.46	73.67	8.82	19.29***	.154
Cardiovascular risk markers						
Systolic blood pressure (mm HG)	119.63	13.66	121.58	12.14	0.11	.001
Diastolic blood pressure (mm HG)	82.07	8.38	79.97	8.05	2.63	.023
Total cholesterol (mmol/L)	4.06	0.88	4.00	0.82	0.08	.001
LDL cholesterol (mmol/L)	2.26	0.69	2.10	0.62	0.43	.004
HDL cholesterol (mmol/L)	1.18	0.32	1.20	0.30	1.29	.013
Triglycerides (mmol/L)	1.49	0.68	1.66	0.84	0.86	.008
HbA1c (%)	5.39	0.32	5.30	0.21	1.86	.017
hsCRP (mg/L)	1.82	1.73	1.06	0.61	8.58**	.080
Mental health						
PTSD symptoms (IES-R)	35.24	23.35	32.84	20.74	0.03	.000
Depressive symptoms (PHQ-9)	10.93	7.79	9.79	6.73	0.01	.000
Anxiety symptoms (GAD-7)	8.93	6.90	8.55	6.33	0.43	.004
Pain (VAS)	25.61	19.43	24.76	22.05	0.30	.003
Quality of life (WHO-5)	12.92	7.23	15.09	6.54	1.51	.013

*N* = 116, because 28 participants had missing data for VO_2_max. VO_2_max, maximal oxygen uptake; LDL, low density lipoprotein.; HDL, high density lipoprotein; HbA1c, glycated hemoglobin A1c; hsCRP, high-sensitivity C-reactive protein; IES-R, 22-item impact of event scale—revised; PHQ-9, 9-item depression scale of the patient health questionnaire; GAD-7, 7-item general anxiety disorder scale; VAS, 5-item visual analogue scale; WHO-5, 5-item quality of life index of the World Health Organization. *p < 0.05.

** *p* < 0.01.

*** *p* < 0.001.

### 3.6 Fitness as a moderator of the relationship between participants’ weight status and health outcomes

As shown in Table 4, compared to normal weight counterparts (46.8%, *n* = 29), participants with overweight/obesity were more likely to have very low fitness levels (79.6%, *n* = 43), *χ*^2^(1,116) = 13.23, *p* < 0.001. Approximately one in 10 participants (9.5%, 11 of 116) was overweight/obese, but was not classified in the lowest fitness category, whereas the portion of overweight/obese participants with “good” or higher fitness was 5.6% (3 of 54). Against our expectations, only one significant interaction effect was found between weight status and fitness level. *Post-hoc* tests with Bonferroni correction showed that overweight/obese participants with very low fitness levels perceived more pain (*p* < .05) than normal weight participants with very low fitness levels. No statistically significant differences were found between the other groups.

**Table 4:** Two-way analyses of covariance (controlled for age and sex) with weight and fitness status as fixed factors, and body composition, cardiovascular risk markers, and mental health as outcome variables.

	Normal weight				Overweight/Obese									
	Very poor fitness (*n* = 29)		Higher fitness (*n* = 33)		Very poor fitness (*n* = 43)		Higher fitness (*n* = 11)		Weight status		Fitness level		Weight status* fitness level	
	*M*	*SD*	*M*	*SD*	*M*	*SD*	*M*	*SD*	*F*	η^2^	*F*	η^2^	*F*	η^2^
Body composition														
Fat mass (%)	24.20	8.03	19.24	6.51	36.15	9.92	30.82	10.09	49.85***	.312	9.83**	.082	0.73	.007
Muscle mass (%)	71.86	8.07	76.60	6.50	60.66	9.48	65.68	9.61	44.87***	.301	10.53**	.092	0.62	.006
Cardiovascular risk markers														
Systolic blood pressure (mm HG)	117.89	13.85	121.77	12.05	120.80	13.56	121.03	12.96	3.81	.033	0.02	.000	0.15	.001
Diastolic blood pressure (mm HG)	80.10	70.03	79.94	8.54	83.40	9.01	80.06	6.72	1.85	.017	1.58	.014	0.31	.003
Total cholesterol (mmol/L)	3.59	0.69	3.93	0.89	4.42	0.86	4.20	0.57	6.86**	.065	0.36	.004	2.55	.025
LDL cholesterol (mmol/L)	1.97	0.47	2.05	0.62	2.48	0.74	2.24	0.63	4.30*	.042	0.12	.001	1.24	.013
HDL cholesterol (mmol/L)	1.17	0.22	1.19	0.32	1.19	0.37	1.23	0.27	0.13	.001	0.94	.010	0.01	.000
Triglycerides (mmol/L)	1.26	0.56	1.66	0.89	1.66	0.71	1.66	0.70	1.55	.016	0.94	.010	0.98	.010
HbA1c (%)	5.27	0.26	5.30	0.20	5.46	0.33	5.32	0.26	3.19	.030	1.24	.012	1.48	.014
hsCRP (mg/L)	1.54	2.04	1.05	0.61	2.01	1.49	1.10	0.65	1.16	.017	6.15*	.060	0.56	.006
Mental health														
PTSD symptoms (IES-R)	31.40	21.89	32.78	21.95	37.65	24.16	33.03	17.57	0.01	.000	0.00	.000	0.17	.002
Depressive symptoms (PHQ-9)	8.36	6.78	10.12	6.98	12.59	8.01	8.82	6.13	0.04	.000	0.11	.001	2.47	.022
Anxiety symptoms (GAD-7)	6.97	6.08	8.60	6.17	10.21	7.17	8.40	7.18	0.00	.000	0.14	.001	1.09	.010
Pain (VAS)	16.12	13.77	26.45	24.26	32.10	20.20	19.20	11.53	0.06	.001	0.00	.000	6.83**	.063
Quality of life (WHO-5)	13.29	7.62	14.45	6.76	12.69	7.05	17.00	5.67	1.16	.011	2.62	.023	0.96	.009

VO_2_max, maximal oxygen uptake; LDL, low density lipoprotein; HDL, high density lipoprotein; HbA1c, glycated hemoglobin A1c; hsCRP, high-sensitivity C-reactive protein; IES-R, 22-item impact of event scale—revised; PHQ-9, 9-item depression scale of the patient health questionnaire; GAD-7, 7-item general anxiety disorder scale; VAS, 5-item visual analogue scale; WHO-5, 5-item quality of life index of the World Health Organization.

* *p* < 0.05.

** *p* < 0.01.

*** *p* < 0.001.

## 4 Discussion

The key findings of the present study can be summarized as follows: First, in a sample of forcibly displaced individuals living in a Greek refugee camp, almost half of the participants (48.0%) were overweight or obese. Second, approximately six out of 10 participants (62.1%) presented with very poor fitness levels, and the percentage of participants with very poor fitness levels was particularly high among overweight/obese participants (79.6%). Third, overweight/obesity was associated with a less favorable body composition and cardiovascular risk profile, whereas poor fitness was associated with a higher percentage of body fat and a lower percentage of muscle mass. Fourth, overweight/obese participants reported a particularly high pain level if they were unfit.

This study addressed four distinct research questions, which will now be discussed in turn. Our first goal was to assess the prevalence of overweight and obesity in a sample of forcibly displaced individuals living in a Greek refugee camp. Our findings show that a large percentage of the participants were overweight or obese (48.0%), whereas underweight was a less prevalent issue (4.0%). This contrasts with prior research among Western Sahara refugees, where a high double burden of both over- and undernutrition was observed (86). Hence, in our study, overweight and obesity were similarly prevalent in forcibly displaced individuals as in the Greek adult population, in which the prevalence of overweight and obesity has been estimated to be 47.5% percent (87). Given the negative health consequences of overweight/obesity (88), this finding is concerning and underscores the importance of learning more about the causes of overweight/obesity in this specific population and making overweight/obesity prevention a key target for health interventions in refugee and migrant populations (41, 89). There are several effective measures to prevent overweight/obesity, including the promotion of healthy eating (e.g., reduction of sugary drinks, snacks, and fatty foods, increase of consumption of fruit and vegetables) and regular physical activity (41, 90). Programs focusing on physical activity should encourage different forms of activities, including sports, exercise, play, and physical activity in everyday life (65). Promoting healthy lifestyles could also focus on the family as a whole in order to increase physical activity or increase healthy eating (91).

Our second goal was to estimate participants cardiorespiratory fitness levels. In this regard, it was surprising that based on age and sex-adjusted norms, almost two thirds of the participants (62.1%) had very poor fitness levels. According to the reference data of the American College of Sports Medicine (84), participants with such fitness levels fall into the lowest fitness category (percentile 1–19). This is critical as previous studies have shown that low cardiorespiratory fitness levels are associated with impaired health and increased risks for both all-cause and disease-specific mortality (92, 93). In future research, it would be interesting to learn more about the underlying reasons for the low fitness level. For instance, gathering longitudinal data could help to find out whether fitness levels decline as a function of time residing in a refugee camp, as there might be little opportunity to be or become physically active.

Our third goal was to examine whether and to what degree normal weight and overweight/obese participants differ in body composition, cardiovascular risk markers, and major mental health outcomes. In agreement with the extensive body of available literature, we found that overweight/obesity was associated with lower estimated VO_2_max (94), higher percentage body fat (95), lower relative muscle mass (96), higher blood pressure (97), higher cholesterol levels (particularly LDL cholesterol) (98), higher HbA1c (99) and higher hsCRP (100). These health parameters are well known to contribute to the development of cardiovascular and other chronic diseases (101). Our study also reinforces previous evidence that BMI is sufficiently (>38% of explained variance) associated with participants’ body composition (95), and may thus serve as a screening tool in a refugee camp setting (102). Opposite to the evidence reported in the international literature (28, 29), no differences were found in the present study between normal weight and overweight individuals in mental health outcomes. One explanation might be that overweight is seen more positively in some cultures (103). This could particularly be true for forcibly displaced individuals who have had an arduous flight, faced many deprivations, and currently live in an environment characterized by resource poverty (104). Such an attitude can represent an important challenge in efforts to prevent overweight and obesity in this specific population. With regard to differences between participants with the poorest vs. higher fitness levels, we found only statistically significant differences in body composition and hsCRP. While this concurs well with existing research (105, 106), the lack of significant associations between fitness level and the other health outcomes might be due to a floor effect associated with the generally low fitness levels in this sample, with only 12.9% reporting “good” (or higher) fitness levels.

Finally, only limited evidence was found that among forcibly displaced individuals living in a Greek refugee camp, participants’ fitness is able to moderate the relationship between weight status and cardiovascular and mental health. This is contrary to the literature on the fitness-fatness paradox (45, 55, 59), suggesting that having adequate cardiorespiratory fitness can mitigate the adverse effects of overweight/obesity on cardiovascular health (107). To a certain degree, our study also questions the notion that cardiorespiratory fitness can be achieved independent of body weight. In our study population, among 54 overweight/obese participants with valid fitness data, only 3 (5.5%) achieved “good” (or higher) fitness levels, which is similar to what has been reported in previous studies (59). The only significant interaction between participants’ weight status and fitness was found for pain, showing that overweight/obese participants with poorest fitness perceived higher pain levels than the other study participants. This is an important finding, as chronic pain is associated with reduced quality of life (108) and increased mental health issues (109). Nevertheless, the results of the present study should be considered preliminary until data are available from studies with larger samples in which there is more variation in cardiorespiratory fitness. With other words, the fact that most participants had very low fitness levels might have complicated the detection of statistically significant interaction effects.

The strengths of the present study are that we examined the association between overweight/obesity and cardiovascular and mental health outcomes in an under-researched population. So far, few studies have assessed cardiorespiratory fitness in forcibly displaced individuals. While this is the first study that examined the fitness-fatness concept in refugees, it should be noted that the group of overweight/obese participants with higher fitness levels consisted of a comparably small number of participants. Moreover, the cross-sectional nature of our data precludes causal interpretation of the relationships. Finally, the results of this study cannot be generalized to the general population of forcibly displaced individuals because this group is characterized by high diversity and also includes individuals who have been granted asylum in the host countries. Furthermore, the living conditions in refugee camps as well as the composition of the resident populations can largely differ within and between countries. Finally, it should be noted that we did not include children, younger adolescents, or elderly people, although overweight and obesity constitutes an issue in these populations, as well.

### 4.1 Conclusions

Our study shows that overweight and obesity are prevalent among residents living in a refugee camp in Greece, posing potential risks to their cardiovascular health. Consequently, public health services should prioritize preventive measures and be well-prepared to address associated diseases. We have also noticed that cardiorespiratory fitness is relatively poor in this sample of forcibly displaced individuals. While efforts are needed to improve the fitness of camp residents, the evidence regarding the feasibility and effectiveness of exercise and sport interventions in refugee settings is still in its infancy. Research efforts in this area should be intensified in order to gain relevant insights and to convince political stakeholders of the importance of this issue.

## Data Availability

The original contributions presented in the study are included in the article/Supplementary Material, further inquiries can be directed to the corresponding author.
